# Effect of HZO Thickness Scaling in the Bilayer Ferroelectric Tunnel Junction

**DOI:** 10.1021/acsaelm.5c00469

**Published:** 2025-05-26

**Authors:** Luca Carpentieri, Thomas Mikolajick, Stefan Slesazeck

**Affiliations:** † 435872 NaMLab gGmbH, 01187 Dresden, Germany; ‡ Chair of Nanoelectronics, 9169 TU Dresden, 01187 Dresden, Germany

**Keywords:** ferroelectric tunnel junctions, hafnium zirconium oxide, thickness scaling, TER, field cycling, depolarization field, retention

## Abstract

This study investigates the effects of ferroelectric thickness scaling in a bilayer-structured ferroelectric tunnel junction. It was found that both the remnant polarization and the transport mechanisms exhibit a correlation with the thickness of the ferroelectric film. While variations in ferroelectric thickness influence the tunneling current in the Off state, the magnitude of the remnant polarization significantly affects the current during the On state. Considering that the On–Off ratio serves as an important figure of merit, an analysis of the optimal memory window is provided, accounting for the impact of reading voltage and cycling conditions. Moreover, investigation of the polarization decay observed at different delay times after the writing reveals the direct correlation between the depolarization field and thickness scaling. Retention studies further indicate that tunneling current decay induced greater vulnerability to the On state, primarily attributed to the asymmetry of the stack structure, which results in imperfect screening of polarization charges. Our investigation into the scaling of ferroelectric thickness emphasizes its critical importance by examining both ferroelectric properties and device performance. These findings indicate that the optimization of FTJ for low operation voltage, long data retention, and high on-current density necessitates a coordinated optimization of the layer stack structure, establishing a direct relationship crucial for the future development of hafnia-based FTJ devices.

## Graphical abstract

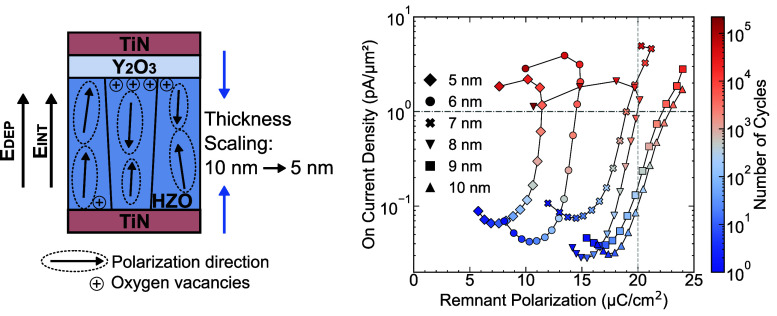

## Introduction

The continuation of Moore’s law is associated with scaling in semiconductor technologies, where doubling the number of transistors every two years results in enhanced performance at reduced cost and power.^1–4^ However, with the recent advent of artificial intelligence (AI),^5^ virtual reality (VR),^6^ and cloud computing,^7^ the necessity for high-speed operations coupled with parallel processing has fostered the development of new computational paradigms outside of the traditional von Neumann architecture and the adoption of innovative nonvolatile device concepts. From this perspective, memory technologies play a pivotal role as new structures, and materials and processing techniques have been developed to withstand and overcome the scaling limits of the SRAM and Flash technologies.^8^ Over the past decade, memristive devices have emerged as promising contenders for future computing architectures. These devices, which utilize resistance changes for data storage, offer unique advantages beyond those achieved by ultimately scaled CMOS.^9^ Their inherent nonvolatility, energy efficiency, and unique switching characteristics make them suitable for a wide range of applications, spanning from nonvolatile memory systems to neuromorphic computing platforms. Ferroelectric materials, which exhibit spontaneous polarization that can be reversed by the application of an electric field, have garnered significant attention following the discovery of an orthorhombic polar phase in hafnium oxide.^10^ Unlike perovskite oxides, which suffer from insufficient thickness scaling and a low coercive field (SBT: 10–100 kV/cm, PZT: 50 kV/cm), hafnia-based ferroelectrics exhibit high remnant polarization when scaled down to a thickness of 10 nm while exhibiting a high coercive field (1–2 MV/cm) independent of the film thickness.^11,12^ Furthermore, their exceptional CMOS compatibility and functionality have enhanced their potential for applications in the upcoming generation of memory devices. Ferroelectric random-access memory (FeRAM), ferroelectric field-effect transistors (FeFETs), and ferroelectric tunnel junctions (FTJ) constitute three different device concepts, in which the coding information is embedded in the electrically alterable polarization direction. While the FeRAM readout scheme is destructive and the FeFET shift of the threshold voltage dictates the polarization direction, in the FTJ, the difference in the tunneling current is used to determine the polarization state during the read operation. Because the changes in the tunneling conductance are linked to the switching of the ferroelectric polarization charges,^13^ the primary limitation encountered in metal-ferroelectric-metal (MFM) stack FTJ is the capability to fabricate high-quality thin films below 3 nm while preserving the ferroelectric properties.^14,15^ Fujii et al.^16^ reported the incorporation of a bilayer structure in FTJ with the addition of a nonpolar dielectric layer, as an additional tunneling layer, to further improve the performance of the device without the constraint of scaling the ferroelectric layer. This novel configuration enables decoupling of the thickness of the ferroelectric layer from the thickness required for tunneling. The dielectric material influences the electron tunneling based on the modulation of the effective tunneling barrier controlled by the direction of the ferroelectric polarization. Consequently, the tunnel electroresistance effect (TER), defined as the ratio of the On and Off currents, can be influenced by both the dielectric thickness scaling and the dielectric material selection. Nonetheless, experimental reports discussing the effect of ferroelectric thickness variations on the performance of double-layer FTJ are limited. In this letter, we report the relationship between the scaling of the ferroelectric thickness and its effect on both the tunneling current and remnant polarization. Specifically, by investigating the behavior of the FTJs with field cycling, we identified an optimum range in which a maximum TER exceeding 10 can be achieved. Retention studies have elucidated the role of the depolarization field and the stability differences between the polarization states arising from the asymmetric interfaces surrounding the ferroelectric material.

## Experimental Details

The FTJ stacks were fabricated on (100) oriented p-doped silicon wafer substrates. A tungsten layer with a thickness of 30 nm was deposited using sputtering (PVD) in an Alliance Concept sputtering tool equipped with a load-lock handling system, maintaining a base pressure of approximately 1 × 10^–7^ mbar. Subsequently, a 10 nm titanium nitride (TiN) layer was sputtered under identical process conditions, employing a Ti target and N_2_ plasma with a flow rate of 4 sccm during the deposition phase. Ferroelectric and dielectric layers were synthesized utilizing the Oxford OpAL system, wherein films were developed through atomic layer deposition (ALD). Specifically, Hf_0.5_Zr_0.5_O_2_ (HZO) was deposited using HfCp­(NMe_2_)_3_ and ZrCp­(NMe_2_)_3_ as metal precursors, with ozone serving as the oxidant source at a temperature of 280 °C. For the HZO films, the ratio of Hf to Zr was maintained at 1:1, while the alternating cycles of Hf and Zr precursors were pulsed for 62 (32) cycles to produce an approximately 10 nm (5 nm) HZO thin film. Regarding the dielectric layer, Y_2_O_3_ was deposited using Y­(MeCp)_3_ as the metal precursor, with ozone as the oxidant source, at a temperature of 300 °C. Following the deposition of TiN, postmetallization annealing was conducted at 450 °C for 300 s in nitrogen atmosphere to crystallize the HZO film. The crystallization temperature was determined by the constraints of the thermal budget for the back-end-of-line (BEOL) integration of CMOS technology. The formation of a capacitor structure with a radius of 100 μm was achieved through the electron beam evaporation of Ti/Pt (10 and 25 nm) using a shadow mask. Ion-beam etching to isolate the top electrode resulted in the fabrication of the entire stack, as illustrated in Figure 1a. Electrical measurements were performed using a Keithley 4200 SCS electrical characterization unit. For the endurance tests, as shown in Figure 1b, each device was subjected to a predetermined number of bipolar field cycles executed with a square waveform at a frequency of 10 kHz followed by a positive-up-negative-down (PUND) waveform at a frequency of 1 kHz. Subsequently, a writing procedure was implemented using square bipolar pulses with a frequency of 10 kHz. The application of a positive bias voltage (Set) to the bottom TiN electrode results in a polarization direction aligned toward the dielectric layer, whereas it orients away from the bottom electrode under a negative bias (Reset). The tunneling current was measured by implementing a DC voltage sweep in the range from 0 to 1.5 V in 50 mV steps. Owing to the ferroelectric thickness variation, the voltage pulse amplitudes were adjusted to maintain a constant field drop across the ferroelectric layer, disregarding any polarization charges, as shown in Figure 1c. An alternative methodology for current readout was employed to determine the retention characteristics of the devices. Specifically, the reading current was acquired through the application of a 500 ms readout pulse, iterated five times, as illustrated in Figure 1d. This approach, which involves adjusting the voltage read amplitude in accordance with the ferroelectric thickness and avoiding a slow DC voltage sweep, facilitates the mitigation of charge trapping during retention measurements. Furthermore, the crystallographic structure of the HZO thin films was examined using grazing incidence X-ray diffraction (GIXRD) with a Cu X-ray source.

**Figure 1:**
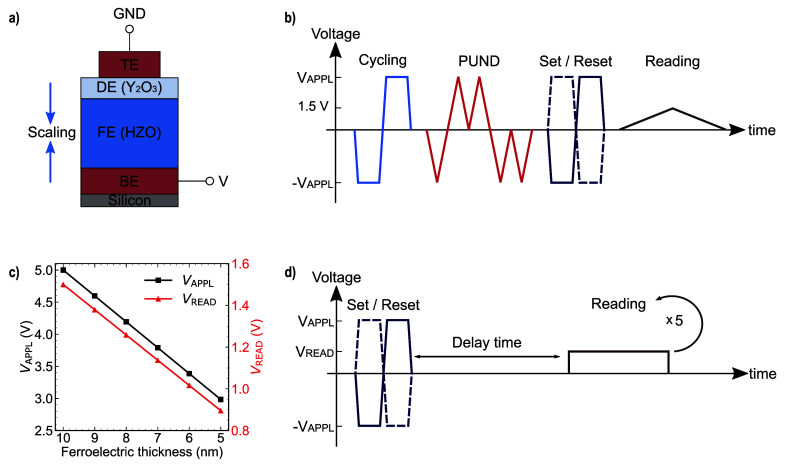
(a) Schematic of the FTJ device featuring HZO and Y_2_O_3_ as ferroelectric and dielectric layers. (b) Endurance measurement pulse sequence for the extraction of the tunneling current as a function of field cycling. (c) Applied voltage amplitude in the endurance test (black line) and reading voltage for the retention test (red line) for the different HZO thicknesses for constant field operation. (d) Retention measurement sequence indicating the application of 5 reading pulse schemes to read out the current.

## Results and Discussion

### Scaling of the HZO Thickness in Double Layer FTJ Devices

Figure 2a shows the diffraction patterns observed after postannealing treatment of six distinct FTJ samples fabricated with ferroelectric thicknesses ranging from 5 to 10 nm. The monoclinic phase, characterized by 2θ peaks at 28.5° and 31.6° for m(111) and m(−111), respectively, exhibited a relatively low intensity, indicating a lower presence of the phase even with decreasing thickness. Conversely, the peak intensity at approximately 30.5°, corresponding to the polar orthorhombic o(111) and tetragonal t(011) phases, exhibits variation among the FTJs. A reduction in peak intensity, along with peak broadening, is observed as the thickness of the ferroelectric layer increases. This observation implies an enhancement in the crystallinity of the film with increasing thickness.^17^ Therefore, it can be deduced that thinner films are likely to display an amorphous or partially crystallized phase. Further insights were gained by examining the gradual shift of the o(111)/t(011) peak with thickness scaling. The rightward shift toward a higher diffraction angle with increasing thickness can be attributed to the increased in-plane tensile strain and, consequently, the transition from the t-phase to the o-phase.^15,18^ Indeed, the ferroelectric characteristics of the devices are likely to be affected by variations in the phase content and crystallographic orientations, which are associated with changes in the thickness of the ferroelectric material. In addition, scaling affects the intensity of the depolarization field.^19^ This field, E_DEP_, which opposes ferroelectric polarization, exhibits strong dependence on both the thickness and material composition of the ferroelectric and interfacial layers:

$$E_{\mathrm{DEP}}=-\frac{Pt_{\mathrm{DE}}}{\epsilon_{\mathrm{FE}}t_{\mathrm{DE}}+\epsilon_{\mathrm{DE}}t_{\mathrm{FE}}}$$ 1

where *t*_DE_ and *t*_FE_ are the dielectric and ferroelectric thicknesses, *P* is the remnant polarization, and ϵ_DE_ and ϵ_FE_ are the relative permittivities of the dielectric and ferroelectric layers, respectively. In our experiment, we propose the integration of a high-*k* dielectric layer, Y_2_O_3_, within the bilayer structure FTJ.^20^ The high dielectric constant of approximately 15,^21,22^ as opposed to the commonly reported values for Al_2_O_3_ and SiO_2_ (with dielectric constants of 9 and 3.9, respectively), is advantageous for reducing both the programming and reading voltages, as well as for mitigating the depolarization field. Before field cycling, the capacitance–voltage characteristics were evaluated at a frequency of 10 kHz (see Section S1 in Supporting Information). Figure 2b illustrates the inverse small-signal capacitance per unit area extracted at ±2.6 MV/cm as a function of the HZO film thickness. From the linear fitting analysis of the measured data, the *y*-intercept provides an estimation of the dielectric capacitance, thereby enabling the extraction of the relative permittivity of the interlayer. Given that the measurements were conducted on FTJs with 1.5 nm Y_2_O_3_ thickness, the calculated dielectric constant was approximately 14.3, which aligns with the values reported in the literature. The results indicate that the properties of the dielectric layer remain unperturbed irrespective of the variation in the ferroelectric thickness. However, as illustrated in Figure 2c, the characteristics of the ferroelectric layer exhibit differences when considering the magnitude of the remnant polarization, *P*_R_, in the pristine state and after 10^3^ field cycles. Notably, when the thickness was increased from 5 to 10 nm, both the positive and negative *P*_R_ values exhibited a 2-fold enhancement. After 10^3^ field cycling, *P*_R_^+^ and *P*_R_^–^ demonstrate increments of 5 and 4 μC/cm^2^, respectively, evidencing the wake-up effect in both FTJs. Although this analysis focuses on the *P*_R_ values derived from PUND measurements, it is important to note that the depolarization field may exert an influence, especially in thin-film samples. A thorough examination of the polarization loss is presented in the retention section. Experimental^23,24^ and theoretical^13,25^ studies have demonstrated that the magnitude of polarization significantly influences the tunneling current by modulating the potential barrier height. Consequently, scaling the ferroelectric layer produces a dual effect: a reduction in the required operation voltage and a potential decrease in tunneling resistance owing to the scaling down of the ferroelectric thickness,^20^ while simultaneously influencing the ferroelectric properties, thus counteracting each other. Moreover, a direct correlation was observed between the ferroelectric thickness and the coercive voltage. As depicted in Figure 2d, for both the pristine and woken-up conditions, a reduction in the coercive voltage, *V*_C_, corresponding to a lower ferroelectric thickness was observed. During electric field cycling, a decrease in the positive *V*_C_ by 0.5 V was observed, which can be attributed to the redistribution of charged defects and depinning of ferroelectric domains.^26–28^ The averaging of the positive and negative *V*_C_ values results in a shift in the polarization hysteresis curve toward more positive bias voltages. This observation suggests the existence of an internal bias field oriented toward the bottom electrodes, which induces the asymmetric stabilization of the polarization state. The validity of this interpretation will be corroborated when investigating retention measurements. Preceding this analysis, our investigation of the device characteristics begins with an assessment of the endurance of the FTJs.

**Figure 2:**
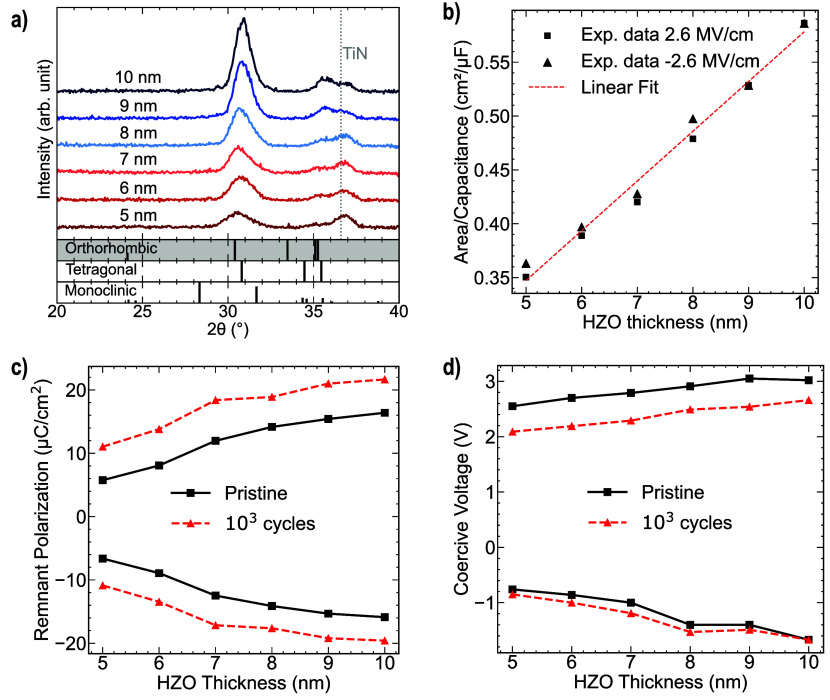
(a) GIXRD patterns of FTJs featuring ferroelectric HZO thickness from 5 to 10 nm. (b) Inverse capacitance per area as a function of HZO thickness. (c) Remnant polarization and (d) coercive voltage as a function of HZO thickness extracted from the PUND measurements in pristine state and after 10^3^ field cycles.

### Tunneling Current over Field Cycling

To investigate the correlation between ferroelectric layer thickness and tunneling current, we conducted experiments on FTJs by applying various numbers of field cycles, spanning from their initial pristine state to the point of device failure. Figure 3a illustrates the evolution of on-current density, measured at a 1.5 V read voltage. Further details regarding the statistical variability among different FTJs, with a focus on remnant polarization and the On and Off tunneling current, are presented in Section S2 of the Supporting Information. A reduction in the ferroelectric layer thickness failed to result in a significant increase in tunneling current. Nevertheless, during field cycling, the tunneling current exhibits a characteristic pattern that remains consistent irrespective of the ferroelectric layer thickness. Figure 3b shows the relationship between the positive remnant polarization and the tunneling current in the On state. The identification of three distinct regions can be observed owning to the inherent impact of the number of field cycles on both parameters. Before 100 field cycles, the stability of the tunneling current, coinciding with the increase in remnant polarization suggests that beyond the wake-up phenomenon observed in the polarization of the ferroelectric HZO material,^29^ a preliminary conditioning process for the FTJ is essential. Within the 100–10^4^ cycle range, both the remnant polarization and tunneling current through the stack demonstrate significant enhancement, with the current exhibiting a 10-fold increase. Consequently, during this interval, a robust positive correlation is observed, indicating this range as the optimal operational window for device functionality. Finally, the fatigue-induced polarization behavior observed after 10^4^ field cycles affects the tunneling current, leading to its eventual plateau (additional information on the remnant polarization as a function of field cycling is reported in Section S3 of the Supporting Information). Further insights can be drawn considering the effect of the ferroelectric layer thickness in Figure 3b. The magnitude of the ferroelectric polarization required to achieve an equivalent tunneling current density across the devices decreases with thickness scaling. Furthermore, when comparing devices with equivalent remnant polarization, a decrease in the ferroelectric layer thickness results in an enhancement of the tunneling current. To provide further insight, Figure 3c depicts the simulated band diagram of FTJs having 10, 7, and 5 nm HZO thickness, considering the value of the remnant polarization after 10^3^ field cycles. As the thickness decreases, the corresponding reduction in P_R_ prevents the Fermi level of the top electrode from exceeding that of the ferroelectric layer conduction band. As a result, the transmission across the stack is expected to decrease despite the reduction in the tunneling distance. However, an increase in the On current is observed despite the lower P_R_ in scaled ferroelectric layer devices. This observation suggests that the current density remains unaffected by thickness scaling, regardless of the magnitude of remnant polarization in the ferroelectric thin film. A different outcome is observed in the off state, as illustrated in Figure 4a. With the exception of the FTJ featuring 7 nm HZO thickness, a decrease in the tunneling current was observed with increasing thickness. Notably, the FTJ with 10 nm HZO thickness exhibited a decline in the current density from 10° to 10^3^ field cycles. We hypothesized that, within this range, the redistribution of charged defects and increased remnant polarization could synergistically contribute to reducing transmission tunneling across the stack. Further investigation is essential to comprehensively elucidate this phenomenon. An analysis of the correlation between the remnant polarization and off-tunneling current, illustrated in Figure 4b, reveals a significant deviation from the trend observed in the On state. In this scenario, the thickness of the ferroelectric layer is a critical factor for stabilizing the current flow during field cycling. Although the increase in remnant polarization, which diminishes the transmission probability across the stack in the off state, persists within 1–10^4^ cycle range for all FTJs, a current plateau prior to 10^3^ field cycles is exclusively observed in devices with ferroelectric thicknesses exceeding 8 nm. This observation suggests that a reduction in the effective tunneling barrier width can facilitate tunneling across the stack, irrespective of the energy-barrier modulation induced by the ferroelectric polarization charges at the interfaces. However, it is important to consider that the distribution and formation of defects within the ferroelectric layer during field cycles could potentially serve as a critical additional factor influencing both ferroelectric polarization and tunneling current. To illustrate the effects of the remnant polarization and ferroelectric thickness in the off state, analogous to the On state, band diagrams for FTJs with HZO thicknesses of 10, 7, and 5 nm are presented in Figure 4c. Upon examination of the dielectric layer, it is evident that polarization-induced band bending reduces with decreasing thickness. This phenomenon results in a reduced effective tunneling barrier, which conversely increases in the On state. Consequently, a reduction in the ferroelectric thickness results in a corresponding decrease in the total barrier width. These results indicate that employing thick ferroelectric films may be beneficial for reducing the probability of tunneling transmission during the Off state of the device. To further investigate the two distinct electrical resistive states, we assessed the tunnel electroresistance effect (TER), defined as TER = *I*_ON_/*I*_OFF_. Given the variations in the tunneling current and remnant polarization during field cycling, we investigated the optimal memory window as a function of the applied read voltage and number of field cycles. Figure 5 illustrates the relationship between the TER and the thickness of the ferroelectric material. Section 4 of the Supporting Information provides a thorough analysis of the current–voltage characteristics after 10^2^, 10^3^, and 10^4^ field cycles for FTJs with HZO thicknesses ranging from 5 to 10 nm, thereby enhancing the understanding of the observed trend. The FTJ with HZO thickness of 5 nm exhibits a maximum TER value of approximately 5 at 10^4^ field cycles when measured with a read voltage of 1.0 V. This observation verifies that a thinner dielectric tunneling barrier layer results in reduced window between the resistance states. This effect is attributed to the increased tunneling transmission probability across the stack during the Off state, which negatively affects the memory window. Analysis of the FTJ with 6 nm HZO thickness reveals a region where the on-state current exceeds the Off state current by a factor of 10. The emergence of a memory window can be attributed to the contribution of remnant polarization. However, examination of the FTJ having 7 and 8 nm thick HZO layers demonstrates how the magnitude of the remnant polarization, as well as the thickness of the ferroelectric HZO, can significantly influence the TER. As both devices exhibit similar remnant polarizations, it can be inferred that the variation in the On and Off currents is primarily influenced by the relative distance that the electrons experience during tunneling. In particular, the device with 7 nm HZO exhibits a maximum TER of approximately 25 at 1.0 V, corresponding to 4 × 10^3^ cycles, whereas the 8 nm thick HZO FTJ achieves a TER of 16 at 2 × 10^3^ cycles and 1.2 V. Consequently, modifications in the ferroelectric thickness not only affect the reading voltage at which the peak TER is detected, but also influence the number of cycles the devices have to undergo for optimum TER. Moreover, the observed reduction in the TER for the FTJ with an 8 nm HZO layer underscores the complex effects of ferroelectric thickness scaling in bilayer-structure FTJs. The concurrent decrease in remnant polarization and increase in conductivity as the stack thickness transitions from 10 to 5 nm places the 8 nm HZO FTJ at a pivotal point. At this stage, the observed decrease in the tunneling current during the On state (Figure 3b) is attributed to a reduction in remnant polarization. Subsequently, there is an increase in the Off tunneling current (Figure 4b), predominantly influenced by the scaling of the ferroelectric layer, which disrupts the linear trend of the TER illustrated in Figure 5. When the scaling exceeds 8 nm, the conductivity is enhanced owing to a narrower barrier width. However, a further increase in the thickness reveals that higher TER values are attainable owing to the more pronounced modulation of the potential profile, which stems from the increased remnant polarization. Our observations indicate TER values of 31 and 43 for the 9 and 10 nm FTJs, respectively, after subjecting the device to 10^3^ field cycles at a read voltage of 1.4 V. Notably, the device with the thickest ferroelectric layer exhibited the highest TER, which correlated with its larger remnant polarization. In addition, a significant memory window can be established after 10^2^ field cycles. However, this requires exceeding the reading voltage beyond 1.2 V. Thus, examining ferroelectric thickness scaling through the investigation of the On and Off current ratio has revealed two primary aspects. A decrease in the memory window toward a lower read voltage was observed with scaling. Moreover, the number of cycles emerges as a critical factor, with our findings indicating a reduction in cycling endurance as the thickness increases, in correspondence with the maximum TER. In summary, the FTJ with the HZO thickness of 10 nm demonstrates the highest endurance and, subsequent to an initial cycling conditioning for 4 × 10^2^ cycles, can be operated with a stable memory window until 10^4^ cycles.

**Figure 3:**
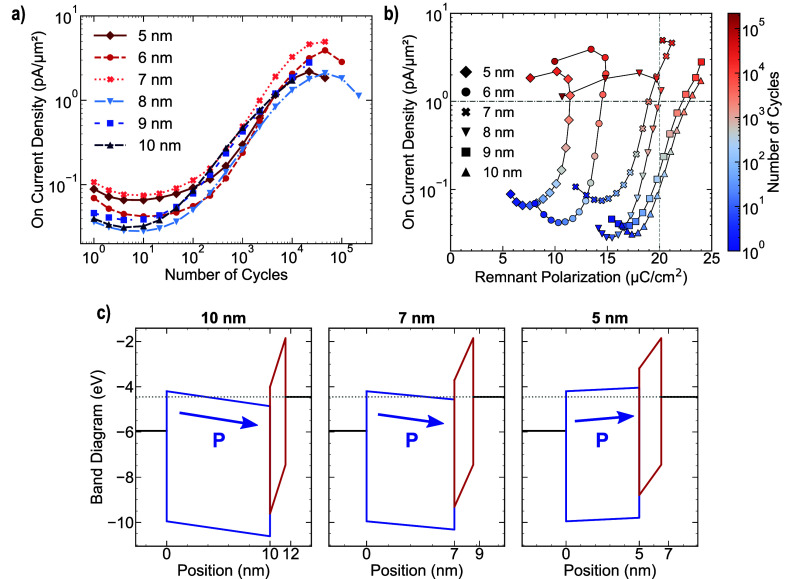
(a) On current density evaluated at *V*_READ_ = 1.5 V as a function of the number of field cycles and the ferroelectric thickness, (b) On current density as a function of the positive remnant polarization and the number of field cycles, (c) band diagram for FTJs having 10, 7 and 5 nm HZO in On-state according to the remnant polarization extracted after 10^3^ field cycles.

**Figure 4:**
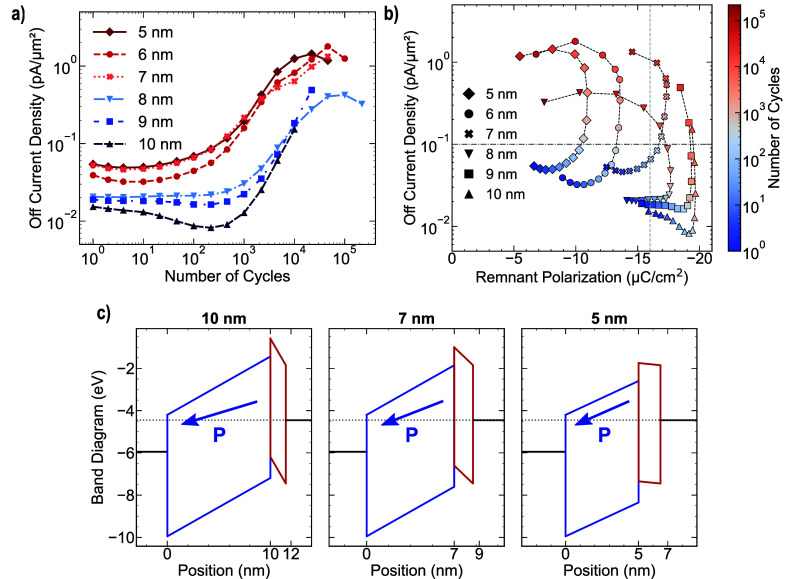
(a) Off current density evaluated at *V*_READ_ = 1.5 V as a function of the number of field cycles and the ferroelectric thickness, (b) Off current density as a function of the negative remnant polarization and the number of field cycles, (c) band diagram for FTJs having 10, 7 and 5 nm HZO in Off-state according to the remnant polarization extracted after 10^3^ field cycles.

**Figure 5:**
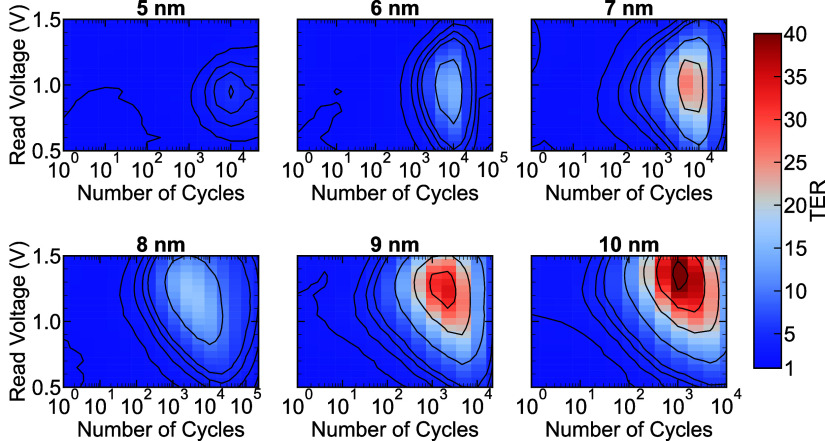
Contour plots showing the dependence of the tunneling electro resistance on number of field cycles and the read voltage for 6 different thicknesses of HZO integrated into a double layer FTJ.

### Short-Term Retention: Polarization and Current

Following the endurance analysis in FTJs, we investigate the retention characteristic of the ferroelectric polarization and tunneling current by considering the impact of both write and read operations. eq 1 indicates that reducing the ferroelectric thickness leads to an increased depolarization field, which is expected to affect ferroelectric polarization stability. To assess this phenomenon, we analyzed the polarization loss over time by monitoring current variations during two set (reset) writing operations across extended delay periods. Figure 6a illustrates the short-time retention behavior of an FTJ featuring 5 nm HZO, including the initial set operation and subsequent set pulses measured at increasing delay intervals ranging from 1 μs to 10 s. In the second writing condition, within a brief time frame, only the contribution of the dielectric current is measured. However, a notable switching current contribution is observed after a specific delay interval between the two pulses (starting at 0.1 ms). This phenomenon can be attributed to the depolarization field, which tends to induce backswitching of ferroelectric domains within the layer. To examine the influence of ferroelectric thickness, we assessed the proportion of polarization loss using eq 2,

$$P_{\mathrm{loss}}=\frac{\int_{0}^{t_{\mathrm{pls}}}I_{\mathrm{Set}}-I_{\mathrm{delay}}}{\int_{0}^{t_{\mathrm{pls}}}I_{\mathrm{Set}}-I_{1\mu s}}$$ 2

where *I*_Set_ indicates the current measured during the initial set pulse and *I*_delay_ represents the current during the subsequent delayed writing pulse. The initial polarization was determined by integrating the difference between the initial set current and the current after 1 μs, because we observed that only the dielectric current component was present within this time frame. This approach allows for the evaluation of polarization switching occurring at progressively longer delay intervals between two set operations. The polarization loss in the ON state, as illustrated in Figure 6b, is presented as a function of delay time and ferroelectric film thickness. As the thickness of the ferroelectric layer decreases, the depolarization field increases, resulting in a more pronounced back-switching effect on the ferroelectric domains. A notable observation is the emergence of a plateau after 10 ms, which occurred irrespective of the thickness of the ferroelectric layer. This phenomenon can be explained by the presence of trapped charges at the interface between ferroelectric and dielectric materials.^30^ This trapping effect plays a role in compensating for the ferroelectric polarization, thus diminishing the effects of the depolarization field. As a result, these findings indicate that the depolarization field has a detrimental impact on device functionality in short time scales. Consequently, the reduction in ferroelectric thickness not only results in a significant decrease in remnant polarization but also, subsequent to the writing process, enables the depolarization field to exert considerable influence. This latter effect induces a reversal of orientation in a subset of ferroelectric dipoles, thereby impacting the reading process. Furthermore, the time delay between programming and reading conditions (exceeding 1 s) indicates that the reduced TER observed in the FTJ with 5 nm HZO is partially compromised by the diminished remnant polarization, which affects the tunneling current in the On state. Conversely, when performing the reset operation, the contribution of the ferroelectric switching current remained undetectable in the second pulse, irrespective of the delay time employed, as shown in Figure 6c. The asymmetry observed in the polarization loss can be attributed to charge trapping at the interfaces, which results in the formation of an internal bias field within the ferroelectric material. As shown in Figure 2d positive bias shift is evident through the averaging of the positive and negative coercive voltages. This internal bias field, *E*_INT_, opposite to the P–V shift, is directed from the top electrode toward the bottom electrode. This result indicates an asymmetric distribution of trapped charges at the ferroelectric interfaces. Shin et al.^31^ demonstrated the diffusion of Y_2_O_3_ into the HZO layer independent of the number of ALD cycles. These findings elucidated the formation of oxygen vacancies due to the substitutional diffusion of Y^3+^ ions into Hf^4+^ or Zr^4+^ sites. Therefore, the uneven loss of polarization between FTJs featuring different ferroelectric thicknesses can be explained by the combined effect of the depolarization field and the formation of positive fixed charges at the ferroelectric-dielectric interface.^30,32^ Figure 6d illustrates that when the polarization is oriented toward the dielectric layer, the ON state experiences destabilization owing to the combined effects of the depolarization and internal bias fields. In contrast, the Off state exhibits a different phenomenon in which polarization stability is attained through the opposing actions of these two fields. Following the analysis of ferroelectric polarization decay under delayed writing conditions, the retention characteristics were investigated by examining the temporal evolution of the tunneling current in both On and Off states. Given that the magnitude of the ferroelectric polarization influences electron transmission across the stack, it is possible to evaluate whether the stability of the Off state induces a corresponding stability in the current. In contrast, the substantial polarization loss observed in thinner films during the On state suggests a rapid decrease in the TER over time. To examine this phenomenon, read-current measurements were conducted at progressively longer delay intervals spanning from 1 s to 10^4^ s. Despite the observed correlation between the number of field cycles and the memory window, our retention measurements primarily focused on analyzing the temporal decay of the tunneling current in devices subjected to 10^3^ field cycles. The temporal progression of the On and Off currents, along with their corresponding TER, is depicted in Figure 7 as a function of the ferroelectric layer thickness. For the FTJ having 5 nm HZO, despite the low current density (<0.01 pA), the On/Off ratio of approximately 3 demonstrates relative consistency as the time interval between the writing and reading conditions increases. This phenomenon can potentially be explained by the decreased polarization, which tends to flatten the potential barrier profile of the dielectric-ferroelectric interface. As a result, the impact of the depolarization field during the initial 0.01 ms writing is so severe that it establishes a steady state, wherein electron transmission across the barrier undergoes minimal alterations over time. In the device featuring 6 nm HZO thickness, the tunneling current in the on-state increases by a factor of 2, which is primarily attributed to the enhancement of the remnant polarization. However, in contrast to the previous scenario, a rapid decrease in tunneling current was observed. While the depolarization field contributes to this phenomenon, an alternative explanation for retention decay involves the accumulation of trapped charges at the interface between the ferroelectric and dielectric layers. These charges potentially counteract the polarization effect within the ferroelectric material, resulting in a retention loss over extended periods.^33–35^ An examination of devices having 7 and 8 nm HZO thick revealed that although remnant polarization remains consistent, the diminished ferroelectric layer thickness may compromise the TER stability over prolonged periods. Moreover, the increase in on-current density associated with thicker ferroelectric layers can give rise to a retention window in which the On/Off current ratio is greater than 10. This phenomenon manifests in device featuring 9 nm HZO within a retention time of 10^3^ s, while 10 nm HZO exhibits a TER value of 22 after 10^4^ s. Therefore, the manifestation of the memory window across extended delay periods can be attributed to the diminution of the tunneling current in the Off state, which exhibits variations in accordance with ferroelectric thickness. The latter focuses on the temporal changes in the On and Off currents, whereas the former explores how the depolarization field influences the ferroelectric polarization relative to the ferroelectric thickness.

**Figure 6:**
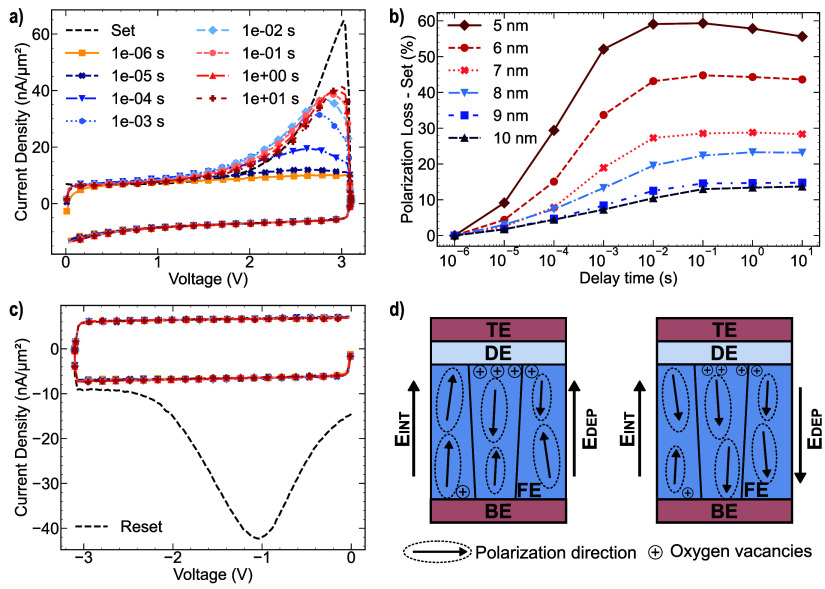
(a) Current–voltage response of the device having 5 nm HZO measured at the initial set operation and after different delay times, (b) percentage of polarization loss according to eq 2 as a function of delay time and ferroelectric thickness, (c) current–voltage response of FTJ featuring 5 nm HZO measured at the initial reset operation with same delay times during set measurement, (d) schematic depictions of the FTJs under set (left) and reset (right) condition with the corresponding depolarization field and internal bias field direction.

**Figure 7:**
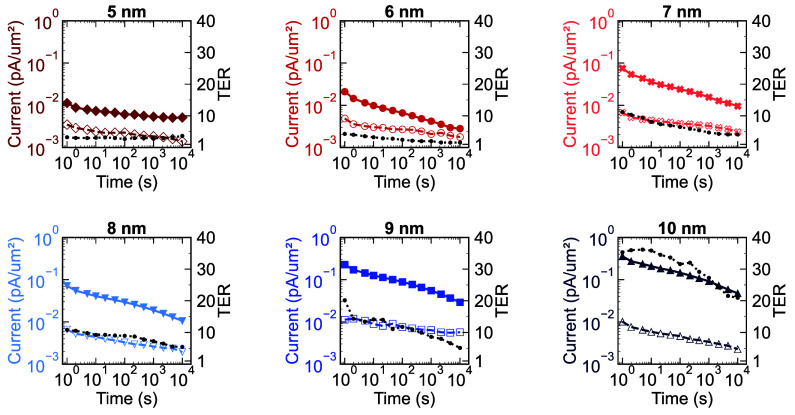
Retention characteristics of FTJs with variable ferroelectric layer thicknesses after 10^3^ field cycles. The graph depicts the On state current density (continuous line), Off state current density (intermittent line), and TER (black lines), demonstrating the evolution of tunneling current and On/Off ratio over time.

## Conclusions

This investigation examines the influence of ferroelectric layer thickness on the characteristics of bilayer FTJ structures. A decrease in the ferroelectric properties was observed as the thickness was reduced from 10 to 5 nm, which was attributed to the diminished orthorhombic phase formation. Given that the modulation of the resistance state is governed by polarization reversal, it follows that not only the thickness variation, but also the magnitude of the remnant polarization can contribute to altering the tunneling transmission probability across the stack. Consequently, we investigated the correlation between the tunneling current and changes in remnant polarization with an increasing number of field cycles. Our observations indicate that the dependence of On-current is predominantly influenced by the more pronounced variation of the potential profile, which is directly correlated with the magnitude of ferroelectric polarization rather than an improved conductivity of a thinner HZO film. Thus, a significant increase in the tunneling current was not observed despite the reduction in the ferroelectric thickness. In contrast, the observed increase in the Off state current with decreasing thickness suggests a direct correlation between the reduction in barrier thickness and current flow. The evaluation of the TER across different cycling conditions and reading voltages indicates that reducing the ferroelectric thickness results in a minor variation in the On and Off ratio at the expense of a large number of field cycles but at a reduced reading voltage. A comprehensive analysis of the retention data was conducted by evaluating current loss over time. The investigation revealed a substantial polarization loss correlated with thickness scaling, attributed to the increasing depolarization field. However, the observed asymmetry in the polarization loss under the set and reset writing conditions can be attributed to the influence of the internal bias field. This phenomenon, resulting from the heterogeneous distribution of trap charges at the interface between the ferroelectric and dielectric materials, influences the asymmetric deterioration of the ferroelectric state and consequently impacts data retention. Furthermore, the similar decay of the tunneling current over time for the On state demonstrates that in addition to the depolarization field, which can affect within short time scales, the accumulation of positive charge trapping at the interface can deteriorate the effect of the ferroelectric polarization. Although the retention data were examined under cycling conditions that yielded the maximum TER among the studied FTJs, the substantial memory window observed after 10^4^ s in the 10 nm case illustrates how increasing the ferroelectric thickness can enhance the device performance. The results of this study demonstrate that thickness reduction in FTJs failed to enhance both the memory window and tunneling current density, primarily because of diminished ferroelectric properties. Consequently, scaling the HZO thickness without altering the remnant polarization would be a target to achieve. Moreover, a reduction in the dielectric thickness facilitates the attenuation of the depolarization field. These observations indicate that the optimization of ferroelectric and dielectric materials is crucial for enhancing device performance.

## Supplementary Material



## Notes

Capacitance–voltage measurements; device-to-device variation in bilayer FTJs: an insight on remnant polarization and tunneling current over field cycling; remnant polarization extracted from PUND measurements as a function of number of field cycles and ferroelectric thickness; and evolution of the On and Off tunneling current after 10^2^, 10^3^ and 10^4^ field cycles (PDF)
