# Supplementary material for: Effect of HZO Thickness Scaling in the Bilayer Ferroelectric Tunnel Junction
Source: ACS Appl Electron Mater. 2025 May 26;7(11):5008–17. doi: 10.1021/acsaelm.5c00469 (PMC12160526; doi:10.1021/acsaelm.5c00469)
Supplement: Supplementary file 1 [file el5c00469_si_001.pdf]

Supporting Information

The effect of HZO thickness scaling in the bilayer ferroelectric tunnel junction

*Luca Carpentieri**, ✤, §, *Thomas Mikolajick*✤, §, *Stefan Slesazeck* ✤

✤ NaMLab gGmbH, Noethnitzer Strasse 64a, 01187, Dresden, Germany.

§ Chair of Nanoelectronics, TU Dresden, 01187, Dresden, Germany.

* Corresponding author e-mail: Luca.Carpentieri@namlab.com

Section S1: Capacitance-Voltage measurements

Considering the variation in the ferroelectric thickness from 10 nm to 5 nm, the voltage applied across the stack was adjusted to maintain a consistent field strength throughout the ferroelectric layer. **Figure S1** presents the capacitance data obtained from the devices in the pristine state and after 10^3 field cycles.

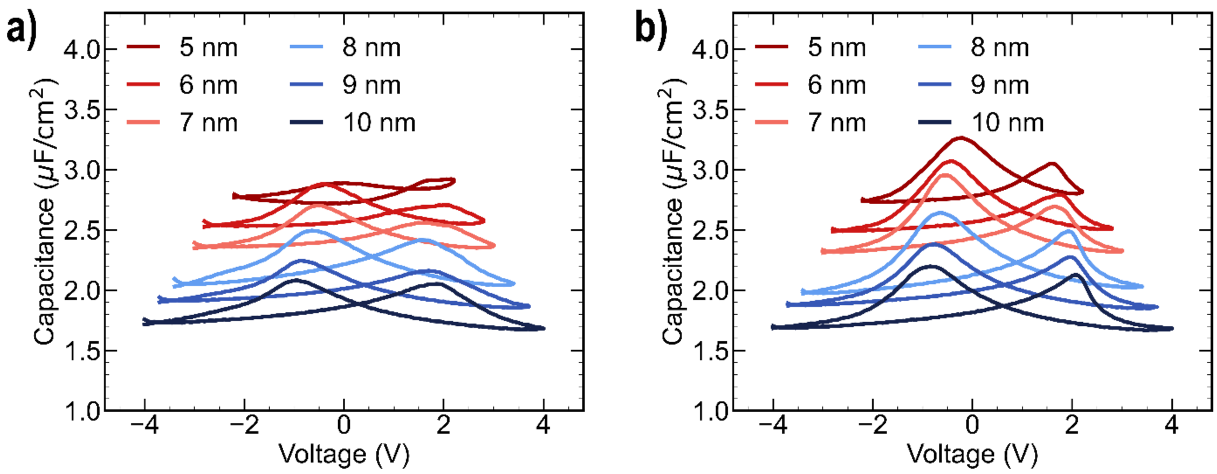

Figure S1. Capacitance per unit of area vs. voltage as a function of HZO thickness extracted in a) the pristine state and b) 10^3 field cycles, respectively.

Section S2: Device-to-device variation in bilayer FTJs: an insight on remnant polarization and tunneling current over field cycling

To substantiate the analysis of tunneling current in ferroelectric tunnel junctions (FTJs) with varying ferroelectric thicknesses, we conduct a statistical examination of remnant polarization and tunneling current in both the On and Off states. This analysis is performed across five distinct cells, which are systematically distributed throughout the sample area. While the scaling of the ferroelectric thickness exerts a significant influence on both the tunneling current and the remnant polarization, we observe only minor variability among the different FTJs.

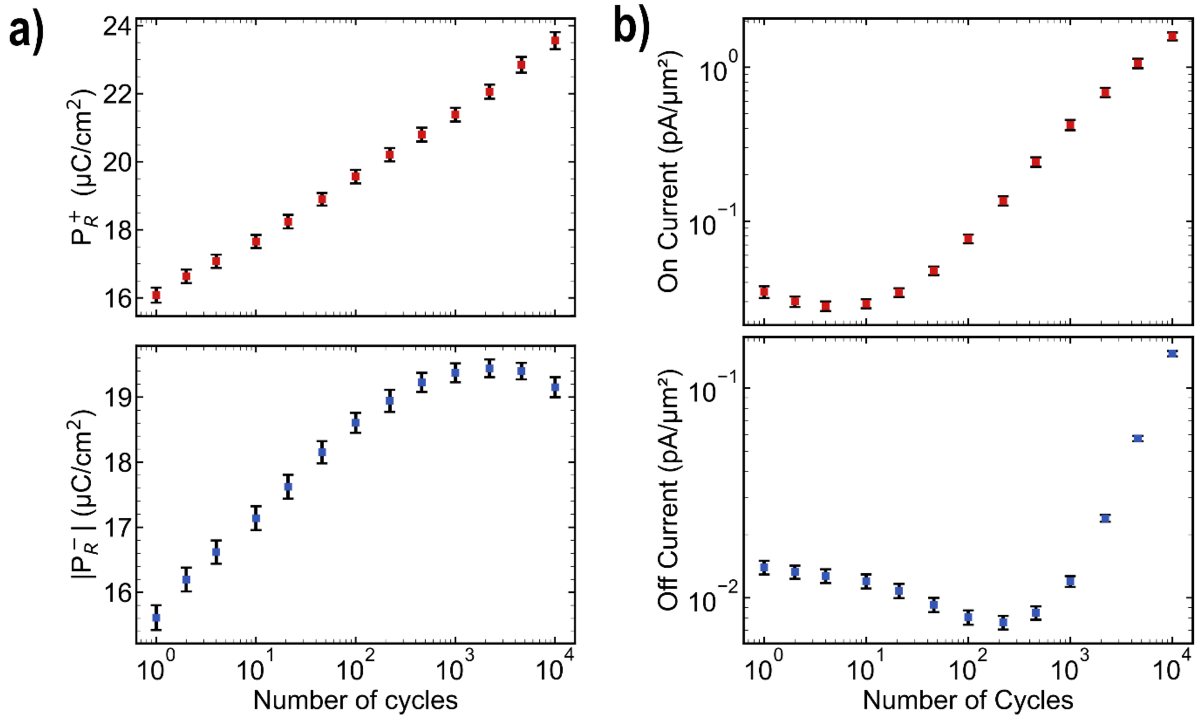

Figure S2. Evaluation of (a) positive and negative remnant polarization and (b) On and Off tunneling current for the FTJ with a 10 nm thick HZO layer. The square symbols denote the mean values, while the vertical error bars represent the standard deviations.

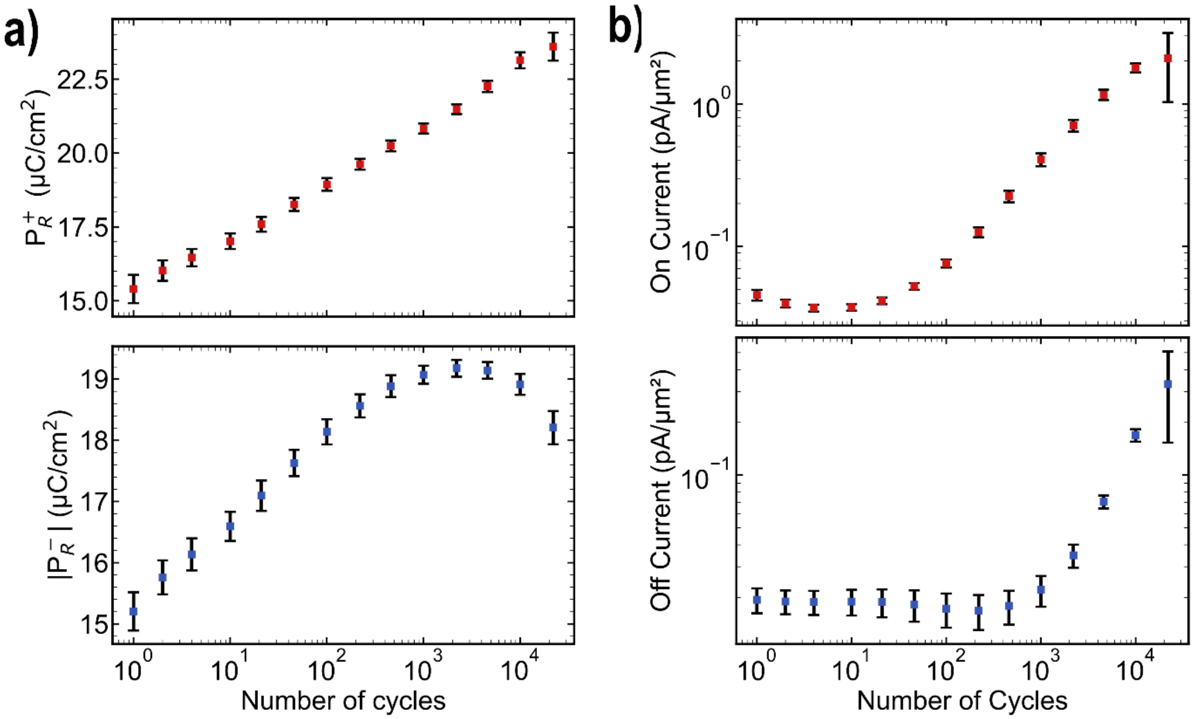

Figure S3. Evaluation of (a) positive and negative remnant polarization and (b) On and Off tunneling current for the FTJ with a 9 nm thick HZO layer. The square symbols denote the mean values, while the vertical error bars represent the standard deviations.

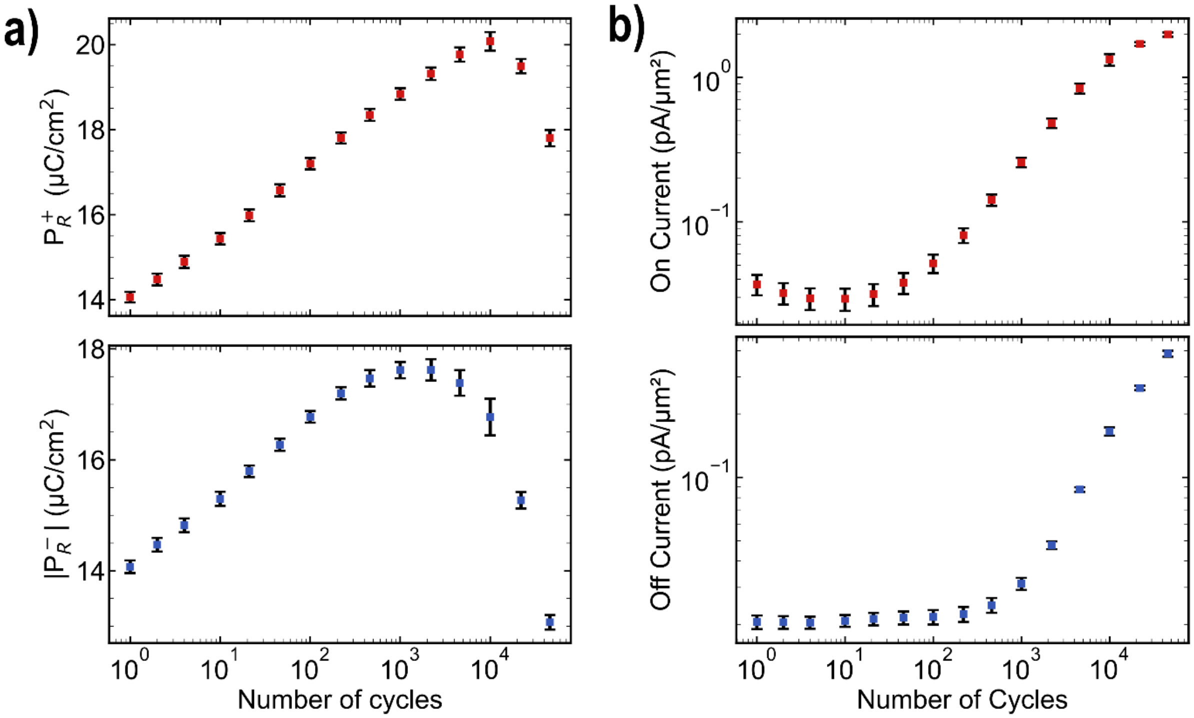

Figure S4. Evaluation of (a) positive and negative remnant polarization and (b) On and Off tunneling current for the FTJ with a 8 nm thick HZO layer. The square symbols denote the mean values, while the vertical error bars represent the standard deviations.

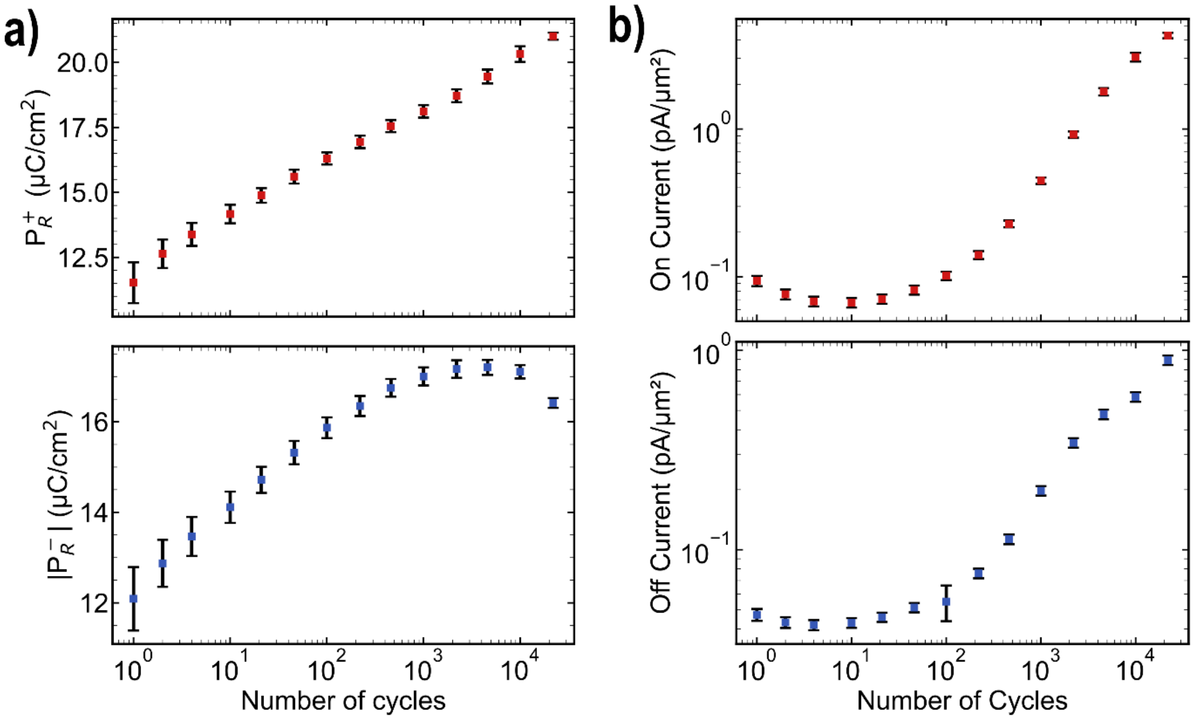

Figure S5. Evaluation of (a) positive and negative remnant polarization and (b) On and Off tunneling current for the FTJ with a 7 nm thick HZO layer. The square symbols denote the mean values, while the vertical error bars represent the standard deviations.

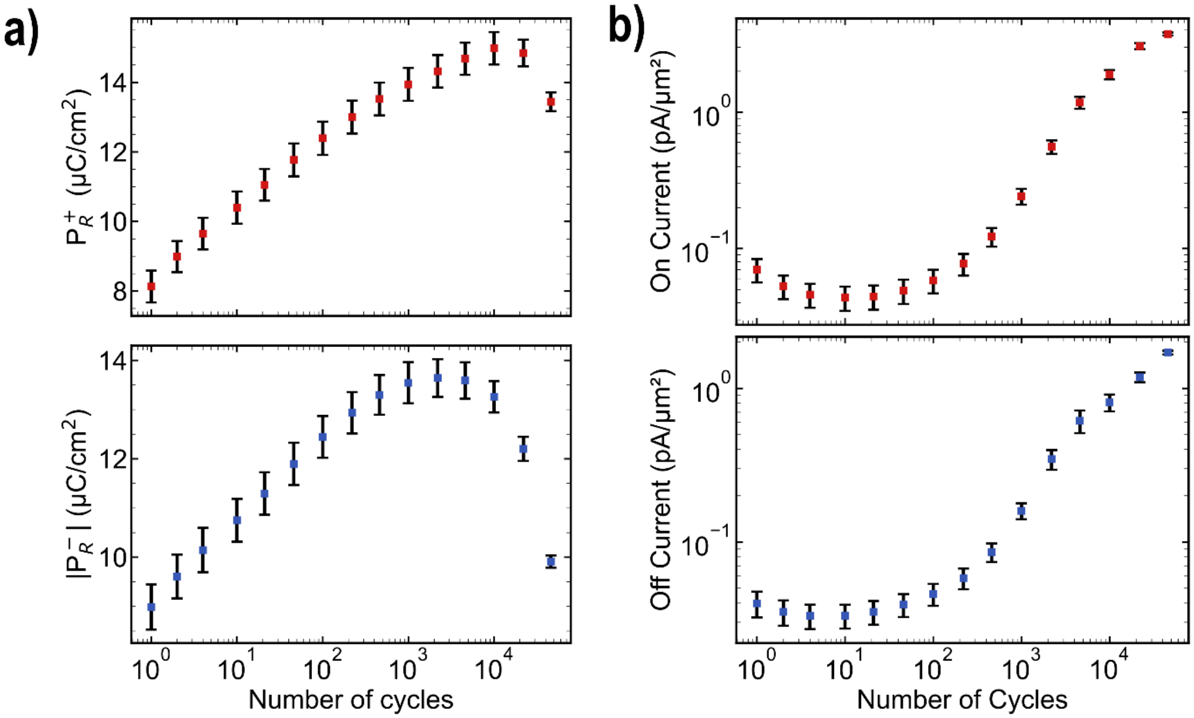

Figure S6. Evaluation of (a) positive and negative remnant polarization and (b) On and Off tunneling current for the FTJ with a 6 nm thick HZO layer. The square symbols denote the mean values, while the vertical error bars represent the standard deviations.

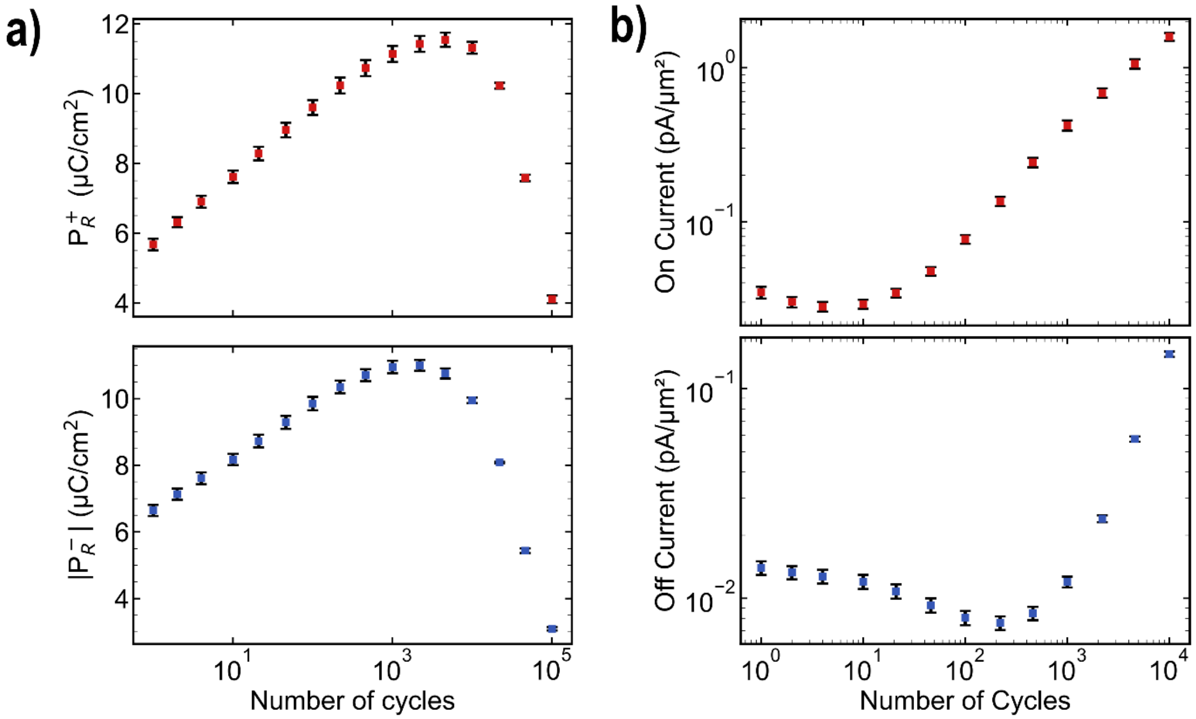

Figure S7. Evaluation of (a) positive and negative remnant polarization and (b) On and Off tunneling current for the FTJ with a 5 nm thick HZO layer. The square symbols denote the mean values, while the vertical error bars represent the standard deviations.

Section S3: Remnant polarization extracted from PUND measurements as a function of number of field cycles and ferroelectric thickness.

Tunneling across the bilayer ferroelectric tunnel junctions has enabled an independent control of the polarization and tunneling properties. This study presents an in-depth examination of the ferroelectric layer, focusing on its behavior in relation to number of field cycles and thickness variations. Consequently, remnant polarization emerges as a crucial parameter for elucidating the properties of FTJ devices.

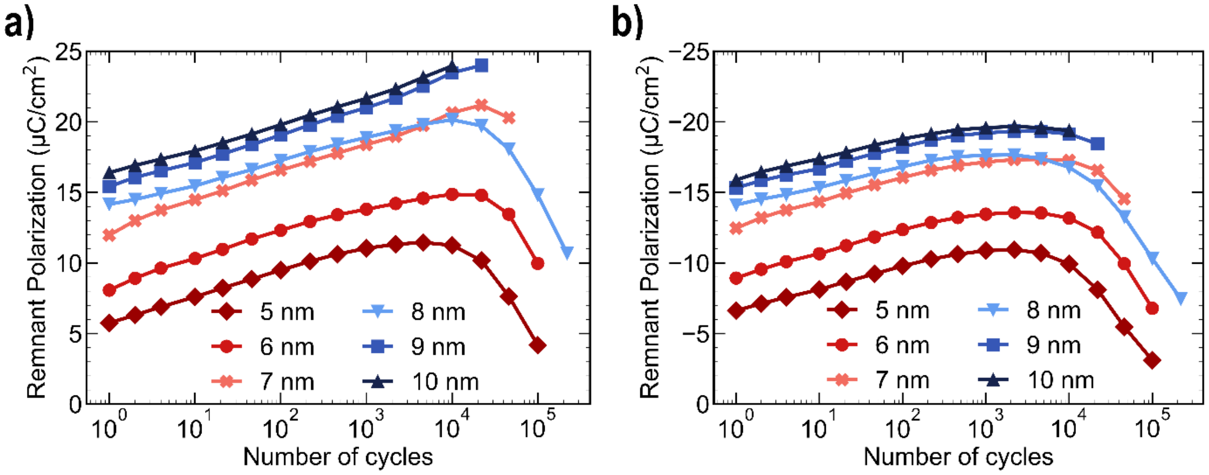

Figure S8. a) Positive and b) Negative remnant polarization as a function of the number of field cycles for 6 different thicknesses of HZO integrated into a double layer FTJ.

Section S4: Evolution of the On and Off tunneling current after 10^2, 10^3 and 10^4 field cycles

In this section, we present the experimental current-voltage characteristics across different FTJs to clarify the extrapolation of the data points depicted in Fig. 3b and Fig. 4b of the manuscript. Considering the progression of tunneling current with the increasing number of field cycles, we analyze the On and Off state behaviors of devices after 10^2, 10^3, and 10^4 field cycles.

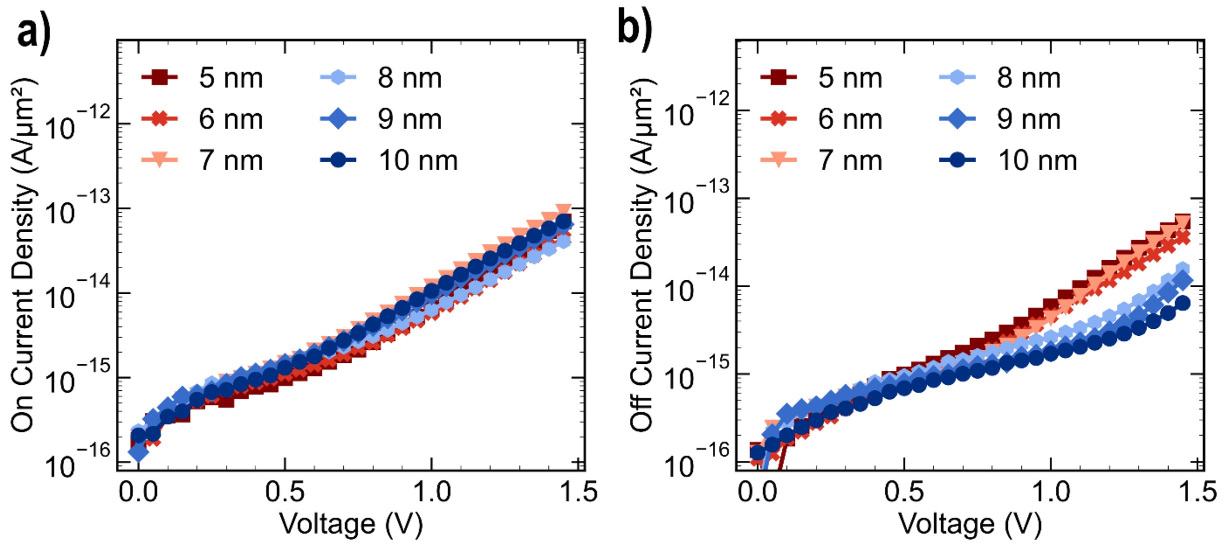

Figure S9. Experimental I-V curve for FTJs featuring HZO thickness from 5 nn to 10 nm reporting the a) On current density and b) Off current density after 10^2 field cycles.

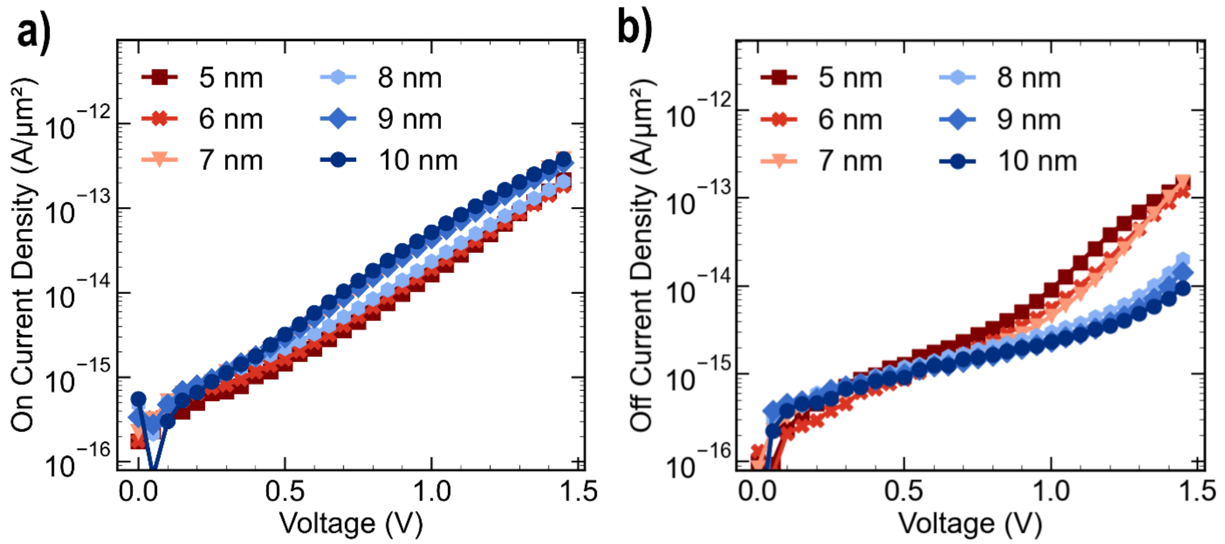

Figure S10. Experimental I-V curve for FTJs featuring HZO thickness from 5 nn to 10 nm reporting the a) On current density and b) Off current density after 10^3 field cycles.

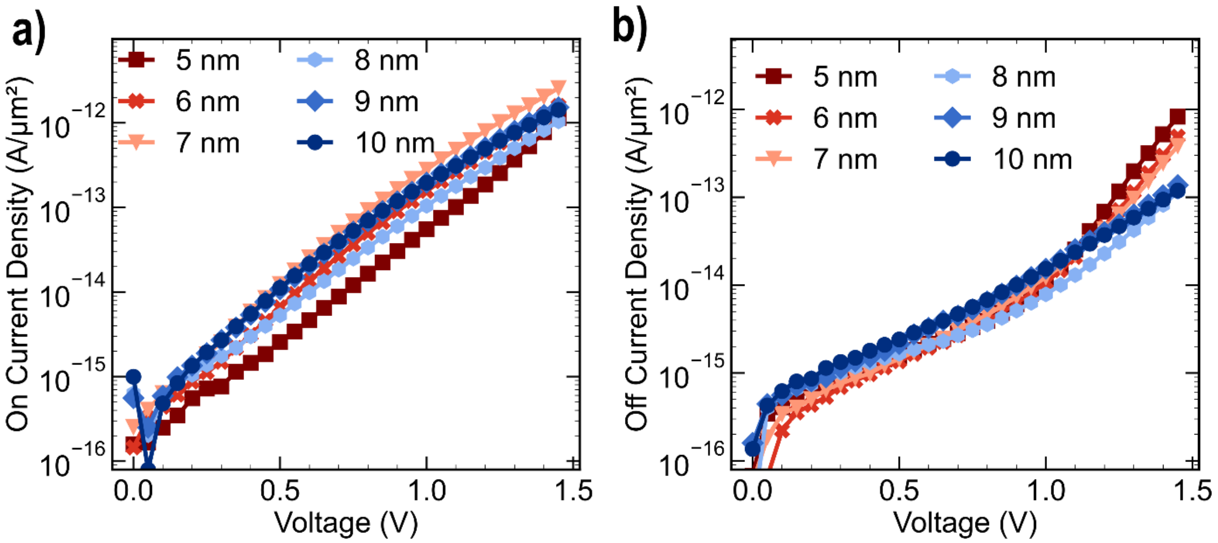

Figure S11. Experimental I-V curve for FTJs featuring HZO thickness from 5 nn to 10 nm reporting the a) On current density and b) Off current density after 10^4 field cycles.
